# Microsatellite based genetic diversity and relationships among ten Creole and commercial cattle breeds raised in Brazil

**DOI:** 10.1186/1471-2156-8-83

**Published:** 2007-12-07

**Authors:** Andréa A Egito, Samuel R Paiva, Maria do Socorro M Albuquerque, Arthur S Mariante, Leonardo D Almeida, Silvia R Castro, Dario Grattapaglia

**Affiliations:** 1 EMBRAPA Recursos Genéticos e Biotecnologia, CP 02372, 70770-970 Brasília, DF, Brazil; 2 Department of Cell Biology, Universidade de Brasília UnB, Brasília, DF, Brazil; 3 Graduate Program in Genomic Sciences and Biotechnology, Universidade Católica de Brasília, 70790-160 Brasília, DF, Brazil

## Abstract

**Background:**

Brazil holds the largest commercial cattle populations worldwide. Local cattle breeds can be classified according to their origin, as exotic or Creole. Exotic breeds imported in the last 100 years, both zebuine and taurine, currently make up the bulk of the intensively managed populations. Locally adapted Creole breeds, originated from cattle introduced by the European conquerors derive from natural selection and events of breed admixture. While historical knowledge exists on the Brazilian Creole breeds very little is known on their genetic composition. The objective of this study was to assess the levels of genetic diversity, phylogenetic relationships and patterns of taurine/zebuine admixture among ten cattle breeds raised in Brazil.

**Results:**

Significant reduction of heterozygosity exists due both to within-population inbreeding and to breed differentiation in both subspecies (taurine and zebuine). For taurine breeds the number of markers that contribute to breed differentiation is larger than for zebuine. A consistently similar number of alleles was seen in both subspecies for all microsatellites. Four Creole breeds were the most genetically diverse followed by the zebuine breeds, the two specialized taurine breeds and the Creole Caracu. Pairwise genetic differentiation were all significant indicating that all breeds can be considered as genetically independent entities. A STRUCTURE based diagram indicated introgression of indicine genes in the local Creole breeds and suggested that occasional Creole introgression can be detected in some Zebuine animals.

**Conclusion:**

This study reports on a comprehensive study of the genetic structure and diversity of cattle breeds in Brazil. A significant amount of genetic variation is maintained in the local cattle populations. The genetic data show that Brazilian Creole breeds constitute an important and diverse reservoir of genetic diversity for bovine breeding and conservation. The genetic data was able to shed light on a number of issues related to the local breeds origin and structure. The Brazilian Creole breeds are all important and viable targets for conservation for they display peculiar traits both phenotypic and of cultural and historical nature that deserve conservation efforts.

## Background

Brazil holds the largest commercial cattle populations worldwide, with over 190 million animals raised both for dairy products and meat [1]. Bovine breeds presently raised in Brazil can be classified into two groups, according to their origin, as exotic or Creole. The group of exotic breeds includes those imported in the last 50 to 100 years, both zebuine and taurine, that currently make up the bulk of the intensively managed populations. Strong directional selection has been shaping these bovine populations in Brazil in the last 40 years mainly through the intensive use of a small number of elite sires in artificial insemination as well as embryo transfer procedures. In spite of the very large census number for some of the most proeminent breeds such as the zebuine Nellore, Gyr and their taurine hybrids, the effective population size has been greatly reduced, although no firm estimate is yet available. A few years back, Georges and Andersson [2] estimated an effective population size of nearly 1,000 for a 10 million animal population of Holstein in the US. It is reasonable to think that with the increased accessibility to assisted reproduction practices, a similar picture is currently the case for all intensively managed bovine breeds in Brazil.

Likewise most European countries and the US, the rapid growth of these commercially proeminent breeds has happened to the expense of the second group of locally adapted, genetically heterogeneous breeds. This group of Creole breeds, also referred as native, local or naturalized breeds includes those derived from the first cattle populations introduced by the European conquerors around 1500. While all other South American countries received only Spanish breeds, due to its peculiar colonial origin Brazil was the only one that received Portuguese breeds [3]. Natural selection acting in remarkably variable environments throughout the country, together with the recurrent events of breed admixture led to the development of Creole breeds adapted to a wide range of environments with outstanding levels of phenotypic variability and improved fitness to local conditions. In the Northeastern regions the Curraleiro breed arised and then moved to the central states of Minas Gerais and Goías. In the Southeastern regions the Junqueira and Franqueiro breeds developed together with the Caracu and Mocho Nacional. In the South the Criolo Lageano breed appeared and in the Pantanal region the Pantaneiro breed.

While historical knowledge has been accumulated on the Brazilian Creole breeds [4-8], very little is known on their genetic composition. Some studies have analyzed sequence variation in the hypervariable regions of the mtDNA and showed, as expected, that both African and European taurine haplotypes are present in American Creole breeds which is consistent with historical records [9,10]. A few reports described preliminary surveys of the genomic polymorphism of some Creole Brazilian cattle breeds, based on low information content RAPD markers that do not allow comparative analyses across independent studies [11,12]. A more systematic and wider scope study based on the "common language" of microsatellite markers is needed to understand the genetic diversity of Brazilian bovine breeds with their peculiar historical origin and present state of endangerment.

In the context of the Guidelines for Development of National Farm Animal Genetic Resources Management Plans [13], the FAO proposed an integrated program for global management of cattle genetic resources using a common set of reference microsatellite markers. Studies of genetic relationships between cattle breeds using a common measuring tool not only provides useful and comparable information on the evolution of breeds to present stage, but also supplies data for a scientifically based development of marker-assisted conservation plans [14,15]. In recent years a number of studies have reported the characterization of cattle breeds throughout the world [16-23]. These studies have progressively used common sets of microsatellite markers thus facilitating comparative surveys of diversity and relationship and the consolidation and analysis of large data sets for multiple breeding, evolutionary and conservation applications.

Following the project proposed for the Animal Genetic Resources by FAO (MoDAD – Measurement of Domestic Animal Diversity)[13] the objective of this study was to assess the levels of genetic diversity, phylogenetic relationships and patterns of taurine/zebuine introgression and admixture among ten cattle breeds raised in Brazil. Diversity was measured at a set of 22 internationally recommended microsatellites, both by FAO and ISAG (International Society of Animal Genetics) to elucidate the genetic relationship of a total of 915 animals belonging to five Creole cattle breeds (Pantaneiro – PAN, Curraleiro – CUR, Criolo Lageano – CRL, Mocho Nacional – MON, and Caracu – CAR) both among them and in comparison with specialized taurine European breeds (Holstein – HOL and Jersey – JER) as well as three major zebuine breeds raised in Brazil (Nellore – NEL, Gyr – GYR and Guzerat – GUZ).

## Results

### Microsatellite markers

A total of 915 animals representing ten Brazilian breeds was analyzed (Table 1). All microsatellite markers showed high polymorphism content in all breeds. A total of 278 alleles were detected over all loci in the 915 animals assayed. Additional file 1 lists all the allele frequency estimates for each microsatellite in each breed. Data will be submitted to the Cattle Diversity Database[25]. The mean number of alleles per locus was 13.2 (ranged between 8 at INRA63 and 23 at TGLA122). Table 2 summarizes the locus specific descriptive statistics for the 22 microsatellite markers, consolidating data across breeds for each *Bos *subspecies (taurine and zebuine) and for both subspecies together. Expected locus heterozygosities in both subspecies and all the breeds combined were nominally larger that the observed heterozygosity for all loci. The only exception was observed at locus ETH3 in the group of zebuine breeds although it did not result in a statistically significant excess of heterozygotes. In the group of taurine breeds only loci INRA63 and HEL1 and in the zebuine group only loci INRA35, INRA37, CSSM66, SPS115, TGLA227, INRA23, ETH3 and BM1824 were found to be in HWE. All other loci displayed deviations from HWE. When all breeds combined were analyzed all loci deviated from HWE (Table 2). The overall loci estimates of inbreeding showed that in both subspecies groups and the consolidated set significant reduction of heterozygosity exists due both to within population inbreeding (F_IS_) and to breed differentiation estimated both under the infinitesimal model (F_ST_) and the step-wise mutation model (R_ST_). A higher estimate of within subspecies inbreeding was seen for zebuine (0.113) when compared to taurine (0.074), although the larger sample size assayed for taurine could be partly accountable for this difference. However when an analysis was carried out on equalized samples sizes of 292 animals per subspecies, results were the same.

**Table 1:** Description of the ten Brazilian bovine breeds studied.

Breed name	Subspecies	Code	# of herds	# males	# females	Total
Caracu	*Bos taurus*	CAR	8	28	49	77
Crioulo Lageano	*Bos taurus*	CRL	1	17	83	100
Curraleiro	*Bos taurus*	CUR	7	43	56	99
Mocho Nacional	*Bos taurus*	MON	4	27	70	97
Pantaneiro	*Bos taurus*	PAN	2	32	64	96
Holstein	*Bos taurus*	HOL	5	25	75	100
Jersey	*Bos taurus*	JER	7	12	42	54
Gyr	*Bos indicus*	GYR	6	22	76	98
Guzerat	*Bos indicus*	GUZ	5	24	76	100
Nellore	*Bos indicus*	NEL	7	42	52	94

**Table 2:** Descriptive statistics of the 22 microsatellite marker loci. Statistics are reported for each *Bos *subspecies separately and overall, consolidating all breeds and all animals: # alleles (N), observed heterozygosity (Ho), expected heterozygosity (He), polymorphism information content (PIC), Wright F-statistics (F_IS_, F_IT_, F_ST_); breed differentiation detected by the marker locus under the step-wise mutation model (R_ST_); statistical significance * = p < 0.05; ** = p < 0.01.

	***Bos taurus *– Taurine breeds (n = 623)**								***Bos indicus *– Zebuine breeds (n = 292)**								**Overall (n = 915)**							
	
**Locus**	**N**	**Ho**	**He**	**PIC**	**F**_IS_	**F**_IT_	**F**_ST_	**R**_ST_	**N**	**Ho**	**He**	**PIC**	**F**_IS_	**F**_IT_	**F**_ST_	**R**_ST_	**N**	**Ho**	**He**	**PIC**	**F**_IS_	**F**_IT_	**F**_ST_	**R**_ST_
**INRA35**	11	0.463	0.597	0.561	0.224**	0.233**	0.077**	0.102	12	0.788	0.830	0.806	0.050	0.065	0.047**	0.028	12	0.569	0.705	0.673	0.193**	0.202**	0.106**	0.119
**HEL9**	13	0.790	0.888	0.877	0.110**	0.115**	0.038**	0.075	13	0.756	0.897	0.886	0.157**	0.163**	0.021	0.035	13	0.779	0.903	0.894	0.137**	0.141**	0.044**	0.056
**INRA63**	8	0.643	0.680	0.622	0.055	0.066	0.083*	0.109	7	0.513	0.582	0.541	0.119*	0.126**	0.023	-0.002	8	0.602	0.732	0.683	0.177**	0.192**	0.177**	0.397
**INRA37**	17	0.771	0.833	0.811	0.074**	0.084**	0.067*	0.097	14	0.781	0.822	0.797	0.049	0.056*	0.022**	0.066	17	0.774	0.843	0.825	0.082**	0.089**	0.067**	0.154
**ILSTS05**	9	0.442	0.601	0.565	0.265**	0.277**	0.111	0.124	9	0.701	0.823	0.802	0.149**	0.156**	0.025**	0.019	9	0.523	0.730	0.703	0.284**	0.295**	0.156**	0.142
**HEL5**	13	0.652	0.895	0.885	0.272**	0.277**	0.045	0.081	13	0.250	0.871	0.856	0.713**	0.714**	0.010	0.052	13	0.536	0.898	0.889	0.403**	0.406**	0.044**	0.136
**ETH152**	10	0.739	0.796	0.771	0.071*	0.078**	0.057*	0.070	10	0.299	0.389	0.377	0.233**	0.238	0.021**	-0.002	10	0.601	0.772	0.742	0.222**	0.236**	0.187**	0.267
**INRA5**	11	0.548	0.719	0.674	0.238**	0.250**	0.102**	0.173	11	0.732	0.836	0.813	0.125**	0.142**	0.054**	0.073	11	0.601	0.774	0.742	0.225**	0.233**	0.108**	0.137
**HEL1**	10	0.738	0.759	0.723	0.028	0.029	0.012*	0.009	10	0.675	0.778	0.747	0.133**	0.139**	0.022	0.082	10	0.718	0.819	0.795	0.124**	0.132**	0.086**	0.045
**CSSM66**	15	0.794	0.877	0.864	0.094**	0.098**	0.032**	0.028	14	0.798	0.826	0.804	0.034	0.041*	0.021**	0.048	15	0.795	0.884	0.873	0.101**	0.106**	0.055**	0.090
**CSSM33**	15	0.686	0.822	0.798	0.166**	0.177**	0.089**	0.057	15	0.754	0.875	0.862	0.139**	0.151**	0.043**	0.006	15	0.707	0.864	0.849	0.181**	0.190**	0.097**	0.251
**CSSM9**	20	0.782	0.858	0.844	0.088**	0.096**	0.059*	0.018	20	0.774	0.874	0.862	0.114**	0.126**	0.042**	0.131	22	0.780	0.900	0.892	0.134**	0.142**	0.092**	0.289
**BM2113**	11	0.793	0.855	0.841	0.072**	0.080**	0.054**	0.068	10	0.734	0.841	0.819	0.128**	0.140*	0.044	0.022	11	0.774	0.865	0.852	0.105**	0.111**	0.063**	0.120
**ETH10**	9	0.695	0.768	0.734	0.094**	0.105**	0.080**	0.123	6	0.607	0.697	0.638	0.129**	0.196	0.230	0.054	9	0.667	0.83	0.808	0.197**	0.214**	0.208**	0.547
**SPS115**	9	0.550	0.609	0.580	0.096**	0.104**	0.059*	0.107	7	0.638	0.700	0.653	0.089	0.112	0.077	0.027	9	0.578	0.663	0.639	0.128**	0.136**	0.096**	0.102
**TGLA122**	23	0.817	0.927	0.921	0.119**	0.123**	0.033**	0.032	19	0.815	0.886	0.875	0.080**	0.085*	0.016	-0.002	23	0.816	0.925	0.920	0.118**	0.121**	0.038**	0.122
**ETH225**	12	0.779	0.855	0.837	0.089**	0.096*	0.051*	0.015	12	0.529	0.664	0.643	0.203**	0.208**	0.020	0.015	12	0.699	0.863	0.848	0.190**	0.200**	0.124**	0.346
**TGLA227**	14	0.755	0.870	0.857	0.133**	0.145**	0.090**	0.187	13	0.384	0.400	0.385	0.042	0.045	0.009	0.044	14	0.635	0.794	0.778	0.201**	0.215**	0.169**	0.370
**TGLA53**	20	0.724	0.870	0.860	0.168**	0.175**	0.053*	0.044	21	0.646	0.787	0.775	0.180**	0.188**	0.027	0.003	21	0.700	0.850	0.840	0.177**	0.181**	0.048**	0.034
**INRA23**	13	0.739	0.785	0.761	0.059*	0.068**	0.066**	0.027	12	0.738	0.781	0.762	0.056	0.064	0.025	-0.000	13	0.738	0.794	0.776	0.071**	0.077**	0.063**	0.018
**ETH3**	11	0.705	0.787	0.765	0.105**	0.114**	0.073**	0.058	9	0.602	0.593	0.536	-0.015	0.002	0.051	0.045	11	0.672	0.770	0.739	0.127**	0.138**	0.122**	0.085
**BM1824**	12	0.696	0.790	0.759	0.119**	0.125**	0.040**	0.047	12	0.693	0.710	0.664	0.024	0.031	0.021	0.015	12	0.695	0.781	0.749	0.109**	0.114**	0.052**	0.044

**Mean**	13	0.6955	0.793	0.769	0.123**	0.131**	0.061**	0.0606	12,23	0.6458	0.748	0.723	0.137**	0.149**	0.040**	0.0549	13.18	0.680	0.816	0.796	0.167**	0.176**	0.098**	0.1861

The contribution of the microsatellite markers for breed differentiation was estimated by the significance of the F_ST _statistics. The number of loci that contributed to breed differentiation varied between the two subspecies with a larger number for taurine when compared to zebuine. Among the taurine breeds only loci ILSTS5 and HEL5 did not contribute to breed differentiation. All other twenty loci had a significant F_ST _with INRA63, INRA5, CSSM33, ETH10 and TGLA227 as the top five loci with the highest nominal values with INRA5 with the highest value at 0.102 (Table 2). In the zebuine group on the other hand, only eight markers contributed to breed differentiation with a significant F_ST _statistics. These were INRA35, INRA37, ILSTS5, INRA5, CSSM66, CSSM33, CSSM9 and ETH152 with the highest significant F_ST _value at 0.054 for INRA5. Interestingly, the nominally highest F_ST _was estimated for locus ETH10 however it was deemed not significant by the jackknife resampling. Estimated values of differentiation due to genetic drift under the step-wise mutation model (R_ST_) were in general more pronounced than by the F_ST _statistics in absolute values. The overall loci estimates of F_ST _and R_ST _were similar in both subspecies groups however R_ST _was much higher that F_ST _when all breeds together were analyzed. The global deficit of heterozygosity when all breeds of both subspecies were combined amounted to 0.176 and the global differentiation among all breeds was estimated by F_ST _at 0.098 and at 0.1861 by R_ST_. Most of this differentiation is likely due to the well known and marked subspecies genetic difference although genetic variation between breed of the same subspecies is also significant (see below).

### Genetic diversity within breeds

Diversity measures for each breed showed a remarkably similar mean number of alleles per locus fluctuating around 8.5. (Table 3). The Creole breeds CRL and PAN were the most diverse populations with the two highest mean allelic richness above 9.0. CAR had slightly less than 8 alleles per locus and was the breed with the smallest allelic richness. Among the zebuine breeds (NEL, GYR, GUZ) the average allele number was very similar, around 8.7 while the two domesticated taurine breeds were less diverse with a smaller average number of alleles slightly above 8.0. Although JER has a smaller sample size than the other breeds this difference did not generate a noticeable reduction of mean allele number when an equalized resampling of 50 animals per breed was analyzed. Average observed and expected heterozigozity ranged from 0.6316 and 0.7409 and 0.7151 and 0.7839 respectively. In all breeds observed heterozygosity values were nominally smaller than the expected ones. Out of the 220 marker by breed HWE tests, 43 were significant, well above the expected 5%. In all breeds at least one microsatellite marker deviated from HWE expectations. MON was the breed where observed and expected heterozigozities were the closest and where the one deviation observed is less than the expected number by chance alone (5% of 22 = 1.1). In all other breeds the number of deviated marker loci cannot be accounted by chance alone. All three zebuine breeds showed several loci deviated from HWE. On the taurine side both the commercial breeds HOL and JER but also the Creole PAN and CUR displayed similar numbers of significantly deviated loci. Highest values of F_IS _were seen for JER followed closely by the three zebuine breeds GYR, GUZ and NEL and the taurine CUR. The average proportion of shared alleles among animals within breeds were similar for all breeds although PAN, MON and CRL had lower values consistent with their highest observed heterozigosities.

**Table 3:** Summary statistics of population genetic parameters for the ten studied breeds. Estimates were obtained averaging over all 22 microsatellites: number of individuals (N); allelic richness, i.e. mean number of alleles/locus (AR); observed heterozygosity (Ho); expected heterozygosity (He); number of Hardy-Weinberg equilibrium deviated loci at p < 0.001 (#HWE); average proportion of shared alleles among animals within breed (APSA) with its standard deviation (SD); * = p < 0.05; ** = p < 0.01.

**Breed**	**N**	**AR**	**Ho (SD)**	**He (SD)**	**F**_IS_	**#HWE**	**APSA (SD)**
**Caracu**	77	7.822	0.6802 (0.0115)	0.7151 (0.0310)	0.0491*	3	0.3839 (0.0780)
**Crioulo Lageano**	100	9.067	0.7102 (0.0098)	0.7625 (0.0292)	0.0682**	3	0.3244 (0.0784)
**Curraleiro**	99	8.838	0.6702 (0.0103)	0.7435 (0.0275)	0.0948**	5	0.3437 (0.0831)
**Mocho Nacional**	97	8.773	0.7409 (0.0097)	0.7763 (0.0225)	0.0454*	1	0.3213 (0.0791)
**Pantaneiro**	96	9.003	0.7229 (0.0100)	0.7839 (0.0184)	0.0775**	4	0.3051 (0.0822)
**Holstein**	100	8.175	0.6847 (0.0103)	0.7406 (0.0232)	0.0755**	6	0.3574 (0.0793)
**Jersey**	54	8.061	0.6316 (0.0146)	0.7142 (0.0314)	0.1210**	4	0.3686 (0.0918)
**Nellore**	94	8.375	0.6454 (0.0109)	0.7220 (0.0318)	0.0957**	6	0.3711 (0.0771)
**Gyr**	98	8.633	0.6357 (0.0108)	0.7235 (0.0326)	0.1196**	5	0.3638 (0.0786)
**Guzerat**	100	8.751	0.6542 (0.0104)	0.7384 (0.0330)	0.1132**	6	0.3469 (0.0763)

### Genetic variation and relationship between breeds

The partitioning of the genetic variation at different levels resulted in small but significant (p < 0.001) between breed proportions of the variation in all structures tested (Table 4). Among the five local Creole breeds variation was the lowest, estimated at 4.43% closer to the value found among the three zebuine breeds, at 4.96%. As expected, highest between groups proportion of variation, almost 17%, was estimated when only the two specialized taurine breeds (HOL and JER) were compared together with the three zebuine breeds. When all breeds were analyzed together, almost 12% of the variation was found among breeds.

**Table 4:** Partitioning of genetic variation at different levels among and within the 10 cattle breeds. Microsatellite marker variation was partitioned by an Analysis of Molecular Variance (AMOVA) under different proposed structures based on subspecies and historical information; Fst values correspond to the AMOVA among population variance; *p < 0.001.

**Structure**	**Source of variantion**	**d.f.**	**Fixation indices**
Local breeds (Creole)	Among populations	4	Fst = 0.04429*
	Within populations	933	
All taurine breeds	Among populations	6	Fst = 0.06202*
	Within populations	1239	
Specialized taurine breeds	Among populations	1	Fst = 0.08309*
	Within populations	306	
Zebuine breeds	Among populations	2	Fst = 0.04959*
	Within populations	581	
Zebuine and taurine specialized breeds	Among populations	4	Fst = 0.16878*
	Within populations	887	
Among all ten breeds	Among populations	9	Fst = 0.11875*
	Within populations	1820	
Taurine vs Zebuine	Among populations	1	Fst = 0.13428*
	Within populations	1828	
Specialized taurine vs Creole vs zebuine	Among populations	2	Fst = 0.11777*
	Within populations	1827	

Estimates of pairwise genetic differentiation based on the infinitesimal model (F_ST_) were all significant after Bonferroni corrections (p < 0.01), indicating that all breeds can be considered as genetically independent entities. Different genetic distance measures were estimated but all showed a very high correlation so that only Nei distance, D_A_, is reported (Table 5). As expected the highest genetic distances were observed between taurine and zebuine breeds such as between JER and NEL (0.3820). Small pairwise distances were observed among the three zebuine breeds. Among the local Creole breeds the lowest distances were observed between PAN and CUR (0.0841). CAR is genetically closer to MON (0.099) which in turn is closer to CRL (0.0861). Among the Creole breeds CRL and PAN were the closest to the zebuine breed NEL suggesting a higher frequency of indicine gene introgression in these two breeds (0.2101 and 0.2320 respectively).

**Table 5:** Pairwise estimates of genetic differentiation and genetic distance among all ten Brazilian cattle breeds. F_ST _estimates above diagonal and Nei genetic distance (D_A_) below diagonal. All estimates of F_ST _were found significant (p < 0.01).

	**CAR**	**CRL**	**CUR**	**GYR**	**GUZ**	**HOL**	**JER**	**MON**	**NEL**	**PAN**
**CAR**		0.084	0.068	0.178	0.193	0.105	0.118	0.047	0.185	0.062
**CRL**	0.153		0.045	0.103	0.117	0.075	0.103	0.034	0.120	0.042
**CUR**	0.124	0.099		0.141	0.157	0.079	0.095	0.041	0.157	0.036
**GYR**	0.326	0.180	0.220		0.033	0.190	0.210	0.125	0.051	0.106
**GUZ**	0.330	0.185	0.232	0.086		0.197	0.216	0.137	0.048	0.122
**HOL**	0.185	0.153	0.175	0.343	0.345		0.083	0.059	0.197	0.077
**JER**	0.209	0.191	0.194	0.368	0.377	0.156		0.076	0.215	0.081
**MON**	0.100	0.086	0.105	0.238	0.254	0.147	0.168		0.138	0.036
**NEL**	0.346	0.210	0.263	0.108	0.103	0.376	0.382	0.275		0.125
**PAN**	0.133	0.088	0.084	0.194	0.199	0.179	0.175	0.088	0.232	

**Figure 1:**
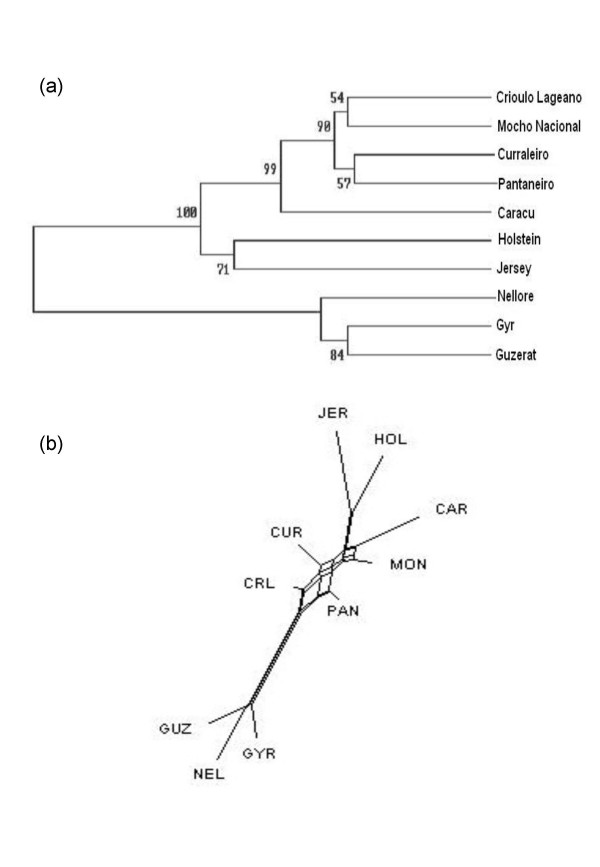
Genetic relationship among ten Brazilian cattle breeds. (a) UPGMA dendrogram and (b) Neighbor-Net graph of genetic relationship among the ten cattle breeds studied based on D_A _genetic distances (Nei, 1983) estimated with 22 microsatellites. Number on the nodes in UPGMA dendrogram are bootstrap values of 10,000 replications.

**Figure 2:**
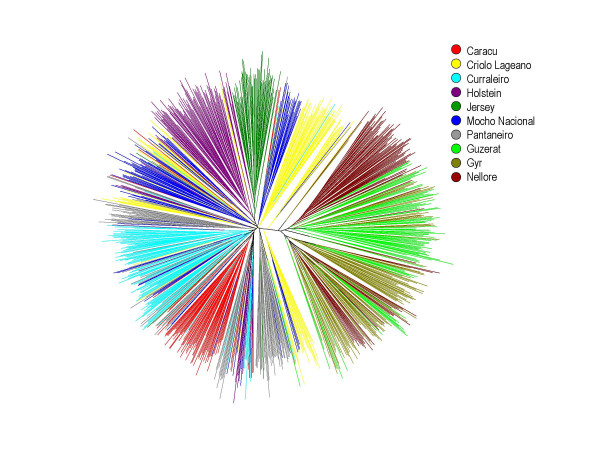
Dendrogram of genetic relationship among all 915 bovine animals. Neighbor-joining tree based on the pairwise genetic distances between all animals estimated by the logarithm of the proportions of shared alleles. Each tip represents a single animal and breeds are distinguished by different colors according to the legend.

The phylogenetic reconstruction from a UPGMA clustering based on the D_A _distance matrix yielded a tree with higher bootstrap values than by the Neighbor Joining method and consistent with known historical and morphological information (Figure 1a). The tree topology was confirmed by the relatively high bootstrap values. Four local Creole breeds CRL, CUR, PAN e MON clustered closer together, with the other three taurine breeds joining in separate branches, JER and HOL closer together. GYR, GUZ and NEL formed a well separated cluster with GYR and GUZ closer together. A Neighbor-Net analysis further corroborates this picture, yielding a better view of the intermediate position of the Creole breeds between the purely taurine and zebuine breeds, and showing a greater proximity of the PAN and CRL breeds to the zebuine group when compared with the other Creole breeds (Figure 1b). An individual-animal-based neighbor-joining dendrogram built from the estimates of allele shared distances among all the 915 individuals shows that the majority of animals within each breed closely assembled in discrete branches, but some exceptions were observed (Figure 2). Taurine and zebuine breeds were clearly segregated in two discrete branches. However while the taurine breeds HOL, CAR and JER formed almost compact sub-branches with few individuals from these breeds misplaced in other breed's clusters, a high frequency of misplaced animals was seen among the Creole breeds and particularly so when looking at the three zebuine breeds particularly so between the GYR and GUZ.

**Figure 3:**
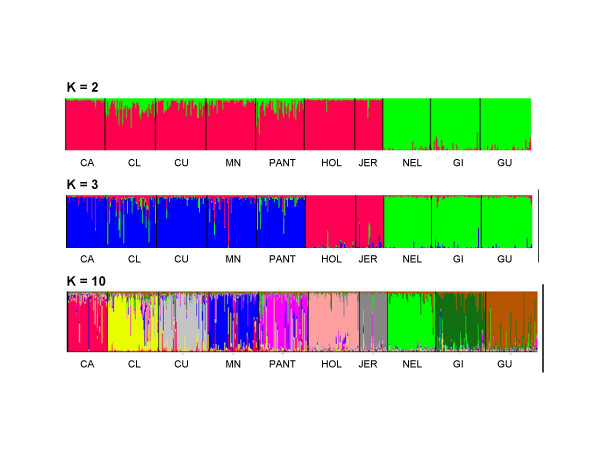
Clustering assignment of the ten Brazilian bovine breeds obtained by STRUCTURE analyses. Each of the 915 animals is represented by a thin vertical line that is divided into segments whose size and color correspond to the relative proportion of the animal genome corresponding to a particular cluster. Breeds are separated by thin black lines. Panels with K = 2 inferred clusters, taurine (red) and zebuine (green) breeds are discriminated; with K = 3, taurine Creole breeds of Iberian origin (blue) are further separated from the specialized taurine breeds (red) and zebuine (green); with K = 10 inferred clusters corresponding to the ten breeds, complex breed admixture patterns can be visualized.

Structure analysis using a Bayesian approach was performed with increasing numbers of inferred populations. Model based clustering at k = 2 resulted in the grouping of the two major subspecies with indications of gene introgression in both directions. With k = 3, local Creole breeds grouped together forming a cluster. It is possible to notice directional matings from the exotic breeds into the local genomes. Based on the values of Q, the most likely k found was k = 10. The diagram clearly shows that admixture has occurred among the local Creole breeds confirming previous indications from the individual-animal dendrogram based on allele shared distances (Figure 3).

## Discussion

To the best of our knowledge this is the most comprehensive report on the genetic structure and diversity of bovine cattle breeds in Brazil, the country with the world largest commercial cattle population and a peculiar mixed composition of both taurine, zebuine and hybrid breeds. The genotype data gathered shows that significant amounts of genetic variation is maintained in the local cattle populations. The Creole breeds CRL, CUR, MON and PAN displayed a distinctly higher allelic richness than both specialized breeds and still nominally higher than the zebuine breeds (Table 3) most likely resulting from the mild selection pressure and a more liberal pattern of herd management. Exception to this trend is the behavior of the Creole breed CAR, the one with the smallest allelic richness and low observed heterozygosity consistent with its unique history of selective breeding. Our results are consistent with the observations of Liron *et al*. [23] when analyzing a group of ten breeds in Argentina and Bolivia that included Creole, taurine and zebuine breeds.

### Microsatellite diversity

The overall average number of alleles observed at each locus, consolidating data for all ten breeds, is above the estimates found in other studies [21,22,26-29]. This larger number can be explained by the relatively larger sample sizes analyzed for the several breeds. Rare alleles, with frequencies below 5% were observed in all breeds in almost every locus (Additional file 1). Estimates of such frequencies below the rule-of-thumb suggested threshold of 5/2n (where n = number of individuals)[30] which corresponds to ~5/200 = 2.5% for most breeds should be seen with caution. Several markers displayed a significant deficit of heterozygotes due to within-population inbreeding in both subspecies and in the combined analysis. Such result has been commonly observed in surveys of bovine breeds in other countries [21,23,27]. The occurrence of null alleles and genotyping errors could also lead to deficiency of heterozygotes. However considering that the estimates of deficit of heterozygotes for the same marker locus varied by subspecies and that the set of microsatellites used has been carefully recommended and broadly used for diversity surveys worldwide [31] this explanation is unlikely.

### Within and between breed genetic variation

The global deficit of heterozygotes (F_IT_) in the sample of 915 animals studied was relatively high, higher than estimates in other studies that involved local breeds both of taurine and zebuine origin [23,29,32]. However it is important to note that in this study Creole taurine breeds were analyzed in conjunction with specialized taurine breeds and zebuine breeds thus deliberately inflating the value of F_ST_. The observed overall reduction of heterozygosity is therefore due in almost equivalent proportions to within-population inbreeding (F_IS _= 0.086) and genetic drift among all ten breeds (F_ST _= 0.098). All breeds displayed a significant reduction in heterozygosity due to non-random matings within populations (Table 3). The three zebuine breeds, JER and CUR had the highest and significant within-population inbreeding coefficients (F_IS_). This result most likely reflects the more intense reproductive management that the zebuine breeds and JER have been subjected to, with the use of a relatively small number of high value bulls as semen donors in assisted reproduction practices.

Two Creole breeds, CAR and MON showed the lowest inbreeding coefficients among all ten breeds. These two breeds have been the subject of concerted efforts to conserve them. MON breed was recovered from a very small number of animals by directed matings coupled to embryo transfer procedures [7]. Furthermore CAR is phenotypically very similar to MON, the only difference being the presence of horns in CAR. The horn removal from CAR animals and matings with MON has led to absorbing crossbreeding of the MON breed by CAR. As the effective population size of MON is still very small, the understanding is that this irreversible breed absorption, although resulting in an uniformization of the two breeds, should ultimately be positive from the practical standpoint as potentially useful alleles will be then conserved in the larger populations of CAR. Such a position has also been advocated as not necessarily undesirable when it constitutes an integral part of the evolution of a breed. Among the five Creole breeds the highest inbreeding was detected in CUR. This was expected as the number of bulls available for this breed is very limited. Current conservation actions for this breed have included the exchange of bulls amongst the few properties that raise these animals as well as expansion of germplasm sampling and cryoconservation [3].

Significant genetic differentiation was observed among all ten breeds estimated both by F_ST _= 0.098 and R_ST _= 0.1861 (Table 2). Similar F_ST _values have been estimated among taurine and zebuine African breeds (F_ST _= 0.06) [29]; 0.112 among seven taurine European breeds [17]; 0.035 among taurine Belgian breeds [33]; 0.107 in a group of northern European breeds [28]; around 0.07 among Iberian and French breeds [19,32] and 0.089 among local taurine Portuguese [22]. In a study similar to ours, when a group of Creole taurine and zebuine breeds from Argentina and Bolivia were analyzed differentiation was estimated at F_ST _= 0.088 and R_ST _= 0.144 [23]. The much higher estimates of differentiation by the R_ST _when compared to F_ST _suggests that differences among breeds involve not only allele frequencies but also allele size differences due to the mutational behavior of microsatellites.

The significance and values of the overall estimates of F_ST _among all ten breeds for the 22 microsatellites are useful indicators of markers that could be powerful tools for breed differentiation. Differentiation of breeds that belong to different subspecies, taurine or indicine, is a relatively trivial task as several markers with a significant F_ST _could easily diagnose the most likely breed as well as the proportions of zebuine and taurine genomes. Within each subspecies however it would be more difficult. In taurine, for example, out of the twenty markers significantly contributing to interbreed differences, markers INRA63, INRA5, CSSM33, ETH10 and TGLA227, the top five ranked by F_ST _values, could be tested for this purpose. In zebuine, only eight markers showed a significant F_ST_, and all of very low value, so that breed differentiation in this subspecies could demand other kinds of markers such as carefully selected and validated ancestrally informative single nucleotide polymorphisms. Both F_ST _and R_ST _estimates within the taurine and zebuine groups taken separately showed a lower differentiation among the three zebuine breeds when compared to the taurine group. The possible explanation resides in the way that these two groups were introduced and are currently managed in Brazil. No specific breed segregation was practiced at the time and all animals coming from the Indies were generically classified as Zebu [6]. Furthermore, the currently existing tens of millions of zebuine animals have resulted in most part from absorbing crossbreeding between indicine bulls and local dams. Very rarely, if at all, are genetically pure herds still available, directly descending from imported animals on both sexes and totally immune to taurine gene flow [34]. Finally only in 1938 the racial standards for zebuine breeds were described and implemented. Until then, all breeds were registered in a single Herd Book of Zebu breed [35].

Four of the five Creole breeds CRL, CUR, MON and PAN displayed a higher allelic richness than all other breeds. The same comparative pattern of genetic variation was observed by Liron *et al*. [23]. The introduction of taurine animals in the American continent was one of the last dispersal movements of bovines in the world. The founder population of the local current Creole breeds was a small groups of Iberian animals that faced a significant selective pressure due to the tropical climate and biotic stresses and an almost extinction due to the introduction of more productive breeds [7]. However an opposing evolutionary force was the admixture with breeds from very diverse geographical origins [3]. The dispersion of these populations to distinct regions following human migrations, together with the very diverse environmental conditions found in a continental country, very mild directional selective pressure and recurrent breed hybridizations, most likely have shaped the current status of genetic diversity of these breeds. Furthermore, in more recent years, introgression from zebuine breeds has also occurred. Only the CAR breed contrasted to this picture showing a reduced observed heterozygosity and allelic richness (Table 3). This is the only Creole breed that has a history of artificial selection and the decline of this breed in the 60's and 70's could have also contributed to this reduction of genetic variation.

### Genetic relationship among breeds and conservation

The partitioning of the genetic variation from an AMOVA also revealed that the largest amount of variation was always found among individuals within breeds, irrespective of the different structures tested (Table 4). Maximum differentiation was found when comparing zebuine and specialized taurine breeds. A very similar pattern of variance partitioning has been seen in several other studies of bovine breeds [19,22,23] where 90% or more of the variation is contained within breeds. Liron *et al. *[23] however, found only 1% of the variation to be due to differences among Argentinean and Bolivian Creole breeds, smaller that the 4% we found between the Brazilian Creole breeds. Although no formal comparative test for significance can be done on these estimates, the nominally higher value might result from two distinctiveness of the Brazilian Creole breeds. First, Brazil was the only country in South America that received Portuguese taurine breeds [3] that have been shown to have both an European and African evolutionary lineages represented by the Brown Concave and Red Convex groups [22]. Second, as will be shown later, some of these Brazilian local breeds have experienced an increased introgression of zebuine genes. It would be interesting to carry out an extensive joint analysis of the local breeds from several countries in South America together with all Iberian breeds to reconstruct a region-wide picture of the patterns of genetic variation

A comparison of autossomal microsatellite, mtDNA haplogroups and Y-chromosome microsatellite haplotypes has shown that for Bolivian and Argentinean Creole breeds significant male mediated zebuine introgression has taken place [23,36]. The expected pattern for Brazil would be an even larger zebuine ancestral genome proportion in the Creole taurine breeds as one moves north, consistent with the introduction and use of zebuine animals for improved adaptation to tropical climates. Such a trend was detected in our study for all Creole breeds analyzed, and particularly so for CRL and PAN that showed the smallest interbreed genetic distances in relation to the three zebuine breeds (Table 5), and from the STRUCTURE analysis, best seen with k = 3 (Figure 3). Several animals of CRL and PAN displayed a discernible amount of zebuine genome and the proximity of these two breeds with the zebuine group was clearly observed in the Neighbor-Net graph. Historical data gathered in the locations where these animals were sampled, do report the presence of Nellore males or their hybrids in the herds. In CUR and MON zebuine introgression was less pronounced and almost none for CAR animals consistent with the history of a more systematic and segregated breeding management of CAR as a taurine breed. Within the zebuine branch, GYR and GUZ breeds are closer together and in the single-animal dendrogram animals of these two breeds are intermingled, consistent with the geographical proximity of their center of origin in India. The STRUCTURE analysis was able to differentiate these two breeds, however a number of animals showed mixed ancestries. Ibeagha-Awemu *et al. *[29] when analyzing a larger set of African zebuine breeds pointed out, in fact, that the model-based clustering approach implemented by the STRUCTURE program cannot effectively discriminate individuals with very closely related genotypes or very low levels of differentiation to their rightful breed without prior population information.

Much controversy has been going and several approaches have been proposed to assess conservation priorities on the basis of molecular markers [19,37]. No attempt was made in this study to define conservation precedence. All Brazilian Creole breeds are important and viable targets for conservation [3]. They are genetically unique and display peculiar traits that deserve conservation efforts. For example, CUR animals are small, low weight, highly adapted to the semi-arid regions of Brazil and able to survive in very harsh conditions with little food and water while displaying marked resistance to several parasites and high fecundity.

## Conclusion

This study reports on a comprehensive study of the genetic structure and diversity of bovine cattle breeds in Brazil. The genetic analysis showed that a significant amount of genetic variation is maintained in the local cattle populations and all breeds studied could be considered as distinct genetic entities. Four of the five Creole Brazilian breeds displayed a markedly higher allelic richness than all other breeds most likely as a result of a combination of natural selection in diverse environmental conditions, mild artificial selective pressure and recurrent breed hybridizations including introgression from zebuine breeds. The genetic data corroborate historical records in that they indicate that variable patterns of breed admixture have occurred since colonial times shaping the current genetic status of the local breeds. Brazilian Creole breeds constitute an important and diverse reservoir of genetic diversity for bovine breeding and viable targets for conservation for they display peculiar traits both phenotypic and of cultural nature. As pointed out by several authors, many other aspects besides the amount and distribution of genetic diversity have to be taken into account when dealing with conservation strategies of livestock species. Historical, cultural and traditional aspects regarding the use of particular breeds are relevant issues. Furthermore one should not forget the fact that directional selection practiced by man has shaped animal genomes in unexpected ways favoring alleles or genes complexes for which the surrogate neutral markers used in diversity surveys are not necessarily fully representative.

## Methods

### Animals

Ten Brazilian bovine breeds were analyzed, involving a total of 915 animals. The breeds studied can be classified into three groups: (a) Taurine Creole breeds (Caracu – CAR; Criolo Lageano – CRL; Curraleiro – CUR; Mocho Nacional – MON and Pantaneiro – PAN); (b) European taurine breeds (Holstein – HOL and Jersey – JER) and (c) Brazilian zebuine breeds (Nellore – NEL; Gyr – GYR and Guzerat – GUZ) (Table 1). For the breeds where pedigree information was available, unrelated individuals for at least three generations were selected. Total genomic DNA was extracted using a routine salting-out procedure [42]. This study followed the legal aspects and rules to which Embrapa is committed and has been approved by the Ethics Committee of Embrapa Genetic Resources and Biotechnology. Moreover, it followed the legal requirements set by the Genetic Heritage Management Council – CGEN of the Brazilian Ministry of the Environment.

### Microsatellite marker typing

Twenty-two microsatellites were amplified by polymerase chain reaction (PCR) in five different multiplex systems where the forward primer of each microsatellite was labeled either with 6-FAM, HEX or NED fluorochromes according to the expected allele size range. Several of these microsatellites have been commonly used by other groups worldwide thus making possible future comparative analysis or consolidation of data sets. The multiplex systems used were: a 7-plex composed by markers INRA35, INRA37, HEL9, HEL5, INRA63, ILSTS5, ETH152 (annealing temperature T_a _= 56°C); a 2-plex of markers CSSM9, CSSM33 (T_a _= 72°C – 60°C, touchdown program); a 2-plex of markers HEL1, INRA05 (T_a _= 56°C); a 5-plex of markers BM2113, ETH10, SPS115, TGLA122, ETH225 (T_a _= 61°C) and a 5-plex of markers TGLA227, TGLA53, INRA23, ETH3, BM1824 (T_a _= 61°C). Microsatellite CSSM66 was amplified alone (T_a _= 61°C) and the PCR product injected together with markers HEL1 and INRA5 before electrophoresis. Only markers CSSM9 [43] and CSSM33 [44] were not included in those recommended for cattle population diversity studies by the MoDAD program of FAO for Management of Farm Animal Genetic Resources. References and primer sequences for the microsatellites used are available in the Cattle Diversity Database [25].

PCR amplified products were electroinjected on an ABI PRISM 3100 Genetic Analyzer (Applied Biosystems) and data collected under virtual filter D using GeneScan 2.0 and Genotyper 2.1 (Applied Biosystems) to declare alleles. An internal size standard labeled with ROX [45] was used for sizing alleles. Genotypes for eight ISAG recommended loci (BM2113, ETH10, SPS115, TGLA122, ETH225 TGLA227, INRA23, BM1824) were calibrated using reference samples genotyped in the 2005–2006 ISAG comparison test (D. Grattapaglia pers. comm.). The AlleloBin software was used to classify observed microsatellite allele sizes into representative discrete alleles using the least-square minimization algorithm of Idury and Cardon [46].

### Data analyses

Allele frequencies were estimated by direct counting. Parameters of locus diversity were estimated for all microsatellite markers in all breeds using the Cervus software [47], including: observed heterozygosity (Ho), expected heterozygosity (He) and polymorphic information content (PIC) Wright's *F*-statistics for each locus were calculated using Weir and Cockerman's method [48] using FSTAT [49]. A significance test on the estimates of Wright's *F*-statistics (F_IT_, F_IS _and F_ST_) for each microsatellite locus were obtained by constructing 95% and 99% confidence intervals based on the standard deviations estimated by jackknifing across populations using FSTAT.

An exact test was used to determine deviations from Hardy-Weinberg proportions and heterozygosity deficiency using the GENEPOP software package [50]. The Markov Chain method [51] was used to estimate unbiased exact P-values. Estimates of genetic variability for each breed (He, Ho with their associated standard error) were calculated using the Excel Microsatellite Toolkit [52]. FSTAT software was used to calculate the allelic richness (AR) standardized for variation in sample size. Breed differentiation was estimated by Wright's *F*-statistics (F_IT_, F_IS _and F_ST_) and the indicative *P*-value was adjusted by a Bonferroni procedure using the same software package [49]. Using breed information different groupings were formed based on their origin (taurine × zebuine) and prior information (Creole × specialized breed). With these definitions, a hierarchical analysis of variance was carried out using an analysis of molecular variance (AMOVA) approach implemented in the ARLEQUIN package [53].

Genetic distances between breeds was estimated by D_A _[54] using DISPAN [55]. The traditional Reynold's distance (F_ST_) was calculated using FSTAT. The log-likelihood G-statistics [56] was used to estimate P-values and the pairwise significance was established after a standard Bonferroni correction [49]. R_ST _[57] was also estimated using the Microsat program. The product moment correlation (r) and Mantel test statistic were computed for pairwise comparisons of distance matrices. A UPGMA (Unweighted Pair Group Method with Arithmetic mean) tree and a neighbor-joining tree were constructed based on D_A _distances using the Dispan package. Bootstrap values were computed over 1,000 replicates. Additionally a Neighbor-Net graph [58] based on D_A _distances was constructed with SplitsTree4 program [59].

The pairwise genetic distances between all individual animals were estimated by the logarithm of the proportions of shared alleles (Dps) [60], using Microsat [61]. The clustering method [62] was used to construct a tree based on the genetic distance matrix using the Phylip package [63] and the result file was entered into TreeExplorer [64] in order to find a suitable graphic display.

Based on genotypes at the 22 marker loci, individual animals were clustered into a given number of populations and assigned probabilistically to clusters inferred with a Bayesian approach implemented by the STRUCTURE software [65]. The tests were done based on an admixture model where the allelic frequencies were correlated applying burn-in period of 50,000 and 500,000 iterations for data collection. Two to fifteen inferred clusters were performed with three independent runs each. Results were entered into the DISTRUCT program [66] to provide a graphic display.

## Authors' contributions

As part of her PhD thesis, AAE participated in the project conception, carried out most of the experimental work, including microsatellite genotyping and data analysis, and drafted the first version of the manuscript. SRP contributed to the statistical analysis. MSMA, STC and LDA helped with DNA extraction and PCR assays. ARS participated in the project conception and design, provided critical information about Creole breeds and helped reviewing the manuscript. As thesis advisor, DG contributed to the design and execution of the experiments and data analysis and to the writing of the final version of the manuscript.

## Supplementary Material

Additional file 1 Distribution of allele frequencies for the 22 microsatellites in the 10 cattle breeds raised in Brazil. Estimates of allele frequencies for the 22 microsatellites markers in the ten Creole and commercial cattle breeds raised in Brazil. Click here for file
